# Coexpression of GITRL confers resistance to Treg cell-mediated immunosuppression to anti-CD19 CAR-NK cells

**DOI:** 10.3389/fimmu.2026.1818610

**Published:** 2026-07-07

**Authors:** Dayane Schmidt, Sima Ebrahimabadi, Mariane Cariati Tirapelle, Camilly Melo Garcia Ferreira, Matheus Henrique dos Santos, Alison Felipe Biggi, Mara Elisama da Silva Januário, Rodrigo Tocantins Calado, Virginia Picanço-Castro

**Affiliations:** Center for Cell-based Therapy (CTC), Regional Blood Center of Ribeirão Preto, University of São Paulo, Ribeirão Preto, São Paulo, Brazil

**Keywords:** Chimeric Antigen Receptor, CAR-NK Cells, B-Cell Malignancy, Treg cells, GITRL, Immunotherapy

## Abstract

**Introduction:**

Regulatory T cells play a pivotal role in shaping immune interactions, limiting antitumor immunity, and impairing the activity of engineered effector cells. Overcoming regulatory T (Treg) cell–mediated immunosuppression is critical to unlock the full therapeutic potential of chimeric antigen receptor (CAR)-cell therapies. Here, we develop “armored” anti-CD19 CAR-NK cells coexpressing GITR ligand (GITRL) (CAR19-GITRL-NK) to both target malignant B cells and locally counteract Treg-mediated suppression. GITR is highly expressed on Tregs, and engagement by its ligand GITRL can modulate their function, providing a rational strategy to reprogram the tumor microenvironment.

**Methods:**

We generated anti-CD19 NK-92 and primary peripheral blood NK (PB-NK) cells with or without GITRL coexpression using lentiviral vectors, and evaluated their functionality and therapeutic potential. CAR19-GITRL-NK-92 cells were analyzed regarding their expansion, metabolism, cytotoxicity, cytokine secretion, degranulation, and *in vivo* antitumor potential. The cytotoxic activity of CAR19-GITRL PB-NK cells was examined, and their growth was measured in the presence of T CD4⁺ cells transduced with FoxP3 expression vector (iTreg-FoxP3) used as a model for Treg cells.

**Results:**

CAR19-GITRL-NK-92 cells displayed superior expansion and enhanced metabolic fitness. Functionally, both CAR constructs increased the cytotoxicity of NK-92 and cytokine secretion against CD19⁺ Nalm-6 and Namalwa cells in vitro. RNA-Seq revealed 54 upregulated and 34 downregulated genes in CAR19-GITRL-NK-92 versus CAR19-NK-92 cells, with enrichment of pathways linked to immune activation, proliferation, chemotaxis, and cytotoxicity, including increased expression of NCR2, CD2, CMKLR1, IRF5, KIT, and PTPN3. *In vivo*, CAR19-GITRL-NK-92 cells achieved superior tumor control and the longest overall survival. In primary NK cells, CAR19-GITRL enhanced killing of CD19⁺ targets, and GITRL expression attenuated iTreg-FoxP3 mediated inhibition in CAR-NK cells, indicating a resistance mechanism to Treg suppression.

**Discussion:**

Collectively, these data position CAR19-GITRL-NK cells as a potent and innovative strategy that couples direct tumor targeting with active undermining of Treg-driven immunosuppression for the treatment of B-cell leukemias and lymphomas.

## 1 Introduction

Chimeric antigen receptor (CAR)-engineered natural killer (NK) cells have emerged as a promising platform for cancer immunotherapy, combining potent cytotoxicity with a favorable safety profile and a low risk of graft-versus-host disease. Clinical trials using CAR-NK cells from different sources are ongoing, and phase I studies have consistently confirmed their safety in patients (1–3). Nevertheless, important challenges remain, particularly related to NK cell expansion, persistence, and optimal CAR design, as well as the ability to overcome the immunosuppressive tumor microenvironment (TME), including T regulatory (Treg) cell–mediated suppression (4–7).

One major strategy to improve CAR-NK cells performance is cytokine armoring, in which cytokines are co-expressed to enhance proliferation, survival, and effector function. Fourth-generation CAR-NK cells incorporating IL-15 signaling show superior expansion and cytotoxicity in preclinical models (8–10), and results from anti-CD19 CAR-NK/IL-15 products demonstrate safety (2). Alternative cytokine configurations, such as co-expression of IL-21 or IL-27, are also being investigated to further modulate NK cell function (11, 12). Together, these approaches illustrate how cell-intrinsic engineering can optimize NK cell fitness, yet they do not directly address the immunosuppressive TME, particularly Treg-driven inhibition of effector cells.

The glucocorticoid-induced TNF-related ligand (GITRL), a member of the TNF superfamily (TNFSF), has recently been used in combination with CAR-T and CAR-γδ T cells, resulting in improved antitumor responses (13, 14). Its receptor, the glucocorticoid-induced TNF receptor-related protein (GITR), is highly expressed on Treg cells and serves as a marker of activated Tregs (15). Although GITR signaling can promote Treg proliferation, multiple studies indicate that its activation can impair Treg suppressive function (16–19). Thus, GITRL co-expression on CAR effector cells represents an attractive strategy to locally modulate Treg activity and counteract immunosuppression within the TME. GITR signaling in NK cells presents contrasting effects, acting as an activator or inhibitor depending on the context (20–22). Nevertheless, its potential as an “armoring” molecule for CAR-NK cells remains unexplored.

In the present work, we evaluated GITRL co-expression as an armoring strategy for CAR-NK cells to overcome Treg-mediated suppression. For that purpose, we co-expressed GITRL on CD28-CD3ζ-based CAR-NK cells to engage GITR on induced Treg cells (iTreg-FoxP3) and locally modulate Treg-mediated immunosuppression. Our data showed that GITRL co-expression did not impair CAR-NK cell proliferation, *in vitro* cytotoxicity, or granzyme expression. RNA sequencing (RNA-seq) revealed the activation of proliferative and immune response pathways. Moreover, *in vivo* antitumor activity of CAR-NK cells was markedly enhanced by GITRL, suggesting that GITRL-expressing CAR-NK cells have improved therapeutic potential against leukemia. In primary NK cells, CAR19-GITRL further enhanced cytotoxicity compared to the CAR19 control, while also protecting the cells from Treg-mediated suppression.

## 2 Methods

### 2.1 Cell culture

The NK-92 cell line, obtained from ATCC (CRL-2407), was cultured using X-VIVO 10 medium (Lonza) containing 5% heat-inactivated human AB plasma (Brazilian Blood Donation Service of the Regional Blood Center of Ribeirão Preto) and 500 IU/mL IL-2 (Clinigen). The tumor cell lines Nalm-6 (CRL-3273), Namalwa (CRL-1432), and K562 (CCL-243), also obtained from ATCC, were cultured in RPMI medium (Thermo Fisher Scientific) with 10% Fetal Bovine Serum (FBS) (Thermo Fisher Scientific). Peripheral blood NK cells and T cells were obtained from peripheral blood mononuclear cells (PBMCs) isolated from leukoreduction filters of platelet donors at the Blood Center of Ribeirão Preto after Ethics Committee approval. NK cells were isolated using the NK Cell Isolation Kit (Miltenyi Biotec), stimulated with 100 IU/mL IL-2 and K562 feeder cells engineered to express 4-1BB ligand and membrane-bound IL-21 (CSTX002), provided by Dr. Dean A. Lee (Nationwide Children’s Hospital/Ohio State University). Primary T cells were obtained with the EasySep Human T Cell Enrichment Kit (StemCell Technologies). All the cell lines tested negative for mycoplasma using the MycoAlertPLUS Mycoplasma Detection Kit (Lonza) (reference value< 1.0). The cell lines underwent short tandem repeat (STR) analysis and were in accordance with the ATCC profiling.

### 2.2 Construction of lentiviral vectors and production of CAR cells

The CAR19-GITRL sequence was developed and provided by Dr. Hiroshi Shiku (WO 2021/020559). For lentivirus production, transient cotransfection of Lenti-X 293T cells was performed using a second-generation system, which included the gene expression cassette of CAR19-GITRL or the control CAR19, the VSV-G envelope plasmid pMG2D, and the lentiviral packaging plasmid psPAX2. The viral particles were prepared as previously described (23). Lentiviral titers were determined using the K562 cell line. NK-92 cells, T cells, and primary peripheral blood NK (PB-NK) cells were transduced with CAR19 or CAR19-GITRL lentiviral particles. NK-92 cells were transduced by spinoculation at a multiplicity of infection (MOI) of 10, in the presence of 8 μg/mL polybrenee and 16 μM BX795. PB-NK cells were transduced with lentiviral particles pseudotyped with the BaEVRless envelope at an MOI of 5, using 6 μM BX795 and 15 μg/mL protamine sulfate. CAR-positive cells were stained with CD19 CAR detection reagent (Miltenyi Biotec) for 10 minutes followed by Biotin-PE or Biotin-APC secondary antibodies (Miltenyi Biotec) staining during 15 minutes and analysis with FACSymphony A1 (BD Biosciences). Sorting of CAR-positive cells was performed with FACSAria Fusion (BD Biosciences).

### 2.3 Metabolic assays

A Seahorse XFe24 analyzer (Agilent Technologies) was used to measure oxygen consumption rate (OCR) and extracellular acidification rate (ECAR) data of NK-92 cells. Following the manufacturer’s instructions, the analyses were conducted with the Glycolysis stress Kit and the T Cell Metabolic Profiling Kit. Cells were plated at a density of 2x10^5^ in Seahorse XF24 cell culture microplates, previously coated with poly-d-lysine (Thermo Fisher Scientific). The cells were incubated for 1h in an incubator without CO_2_ at 37 °C in the assay medium before analysis. For glycolysis analysis, 10 mM glucose, 1 µM oligomycin, and 50 mM 2-deoxyglucose (2DG) were serially injected. As for the mitochondrial respiration analysis, 1,5 µM oligomycin, 2,5 µM BAM-15, and 0,5 µM rotenone/antimycin A (Rot/AA) were added sequentially.

### 2.4 Cytotoxicity assays

*In vitro* cytotoxic evaluation was performed against Nalm-6, Namalwa, and K562 cells. Tumor cells were labeled with PKH67 (Sigma-Aldrich) and co-cultured with NK-92, CAR19-NK-92, or CAR19-GITRL-NK-92 cells for 5h at effector to target (E:T) ratios of 2:1 and 4:1. For co-culture with PB-NK cells, E:T ratios of 1:1 and 4:1 were used. Data were acquired with FACSymphony A1 (BD Biosciences), and the percentage of cytotoxicity was determined with the following equation:


$$\text{Cytotoxicity }\left(\%\right)\;=\;\frac{100\;\times \;\left(\text{co‐culture death }-\text{ spontaneous death}\right)}{\left(100\;-\text{ spontaneous death}\right)}$$

For degranulation analysis, cells were co-cultured at a 2:1 ratio. Anti-CD107a PE (BD) was added to each well, followed by 2 nmol/µL monensin (Sigma Aldrich) after 1 hour. The cells were then incubated for a total of 5 hours. Before analysis on the FACSymphony A1, each sample was stained with anti-CD56 BV785 (BD Biosciences) for 15 minutes.

Co-culture supernatants were used for analysis of IFN-γ and TNF-α levels using BD OptEIA™ Human IFN-γ ELISA and BD OptEIA™ Human TNF-α ELISA (BD Biosciences).

### 2.5 RNA-Seq

CAR19-NK-92 cells and CAR19-GITRL-NK-92 cells were co-cultured with the Nalm-6 cell line at a 4:1 (Effector: Target) ratio for 24h. RNA was isolated from two biological replicates using the RNeasy kit (Qiagen), following the manufacturer’s protocol. RNA-Seq was conducted by LC Sciences (Houston, TX, USA). Briefly, a Poly(A) RNA sequencing library was prepared following Illumina’s TruSeq-stranded-mRNA protocol. Poly(A) tail-containing mRNAs were purified using oligo-dT. Quality control analysis and quantification of the sequencing library were performed using Agilent Technologies 2100 Bioanalyzer High Sensitivity DNA Chip (Agilent Technologies). Paired-end sequencing was performed using NovaSeq 6000 (Illumina). Differential expression analysis of mRNAs was performed using StringTie to calculate fragments per kilobase million (FPKM). Differentially expressed mRNAs were identified using edgeR, applying a statistical significance threshold of p < 0.05. The RNA-Seq data are available in the NCBI Gene Expression Omnibus (GEO) under the accession number GSE307972.

### 2.6 *In vivo* B-cell leukemia model

For *in vivo* anti-tumoral evaluation, Nalm-6 cells stably expressing luciferase (Nalm-6-luc) were generated via lentiviral transduction and subsequently selected with puromycin at 1 µg/mL. Eight-weeks-old NSG mice of both sexes were intravenously injected with 1×10^4^ Nalm-6 luc cells (Day 0). 4x10^6^ NK-92, CAR19-NK-92, or CAR19-GITRL-NK-92 cells were injected retro-orbitally on days 1, 7, 10, 13, and 16. Tumor burden was surveyed by bioluminescence using IVIS Spectrum *In Vivo* Imaging Systems (Perkin Elmer) after intraperitoneal injection of 150 mg/kg of D-luciferin (Perkin Elmer). All procedures adhered to applicable guidelines for animal care, and animal experiments were approved by the responsible government committee in Brazil (n. 012/2021).

### 2.7 Treg suppression assay

CD4^+^ T cells were transduced with a lentiviral vector expressing FoxP3 under the control of the EF1-α promoter. The cells were spinoculated and cultured with AIM-V medium supplemented with 10% FBS and 200 IU/mL IL-2. The detection of the transcription factor FoxP3 was performed using the Human FoxP3 Buffer Set (BD Biosciences), according to the manufacturer’s instructions. The kit allows nuclear permeabilization which was followed by FoxP3 staining with anti-FoxP3 PE (BD Biosciences). The generated iTreg-FoxP3 cells were co-cultured with TCD4^+^ control at 1:1 ratio in the presence of anti-CD3/CD28 beads during 48h. The co-culture of iTreg-FOxP3 with CAR-PB-NK cells included K562-41BBL-IL21mb feeder cells. Cell numbers were quantified using Countess 3FL Automated Cell Counter.

### 2.8 Statistical analysis

All statistical analyses were performed using GraphPad Prism 8.0. The statistical tests applied are indicated in the figure legends. A p-value of <0.05 was considered statistically significant. ****p<0.0001; ***p<0.001; **p<0.01; *p<0.05.

## 3 Results

### 3.1 Efficient generation of CAR-NK-92 cells coexpressing GITRL

**Figure 1:**
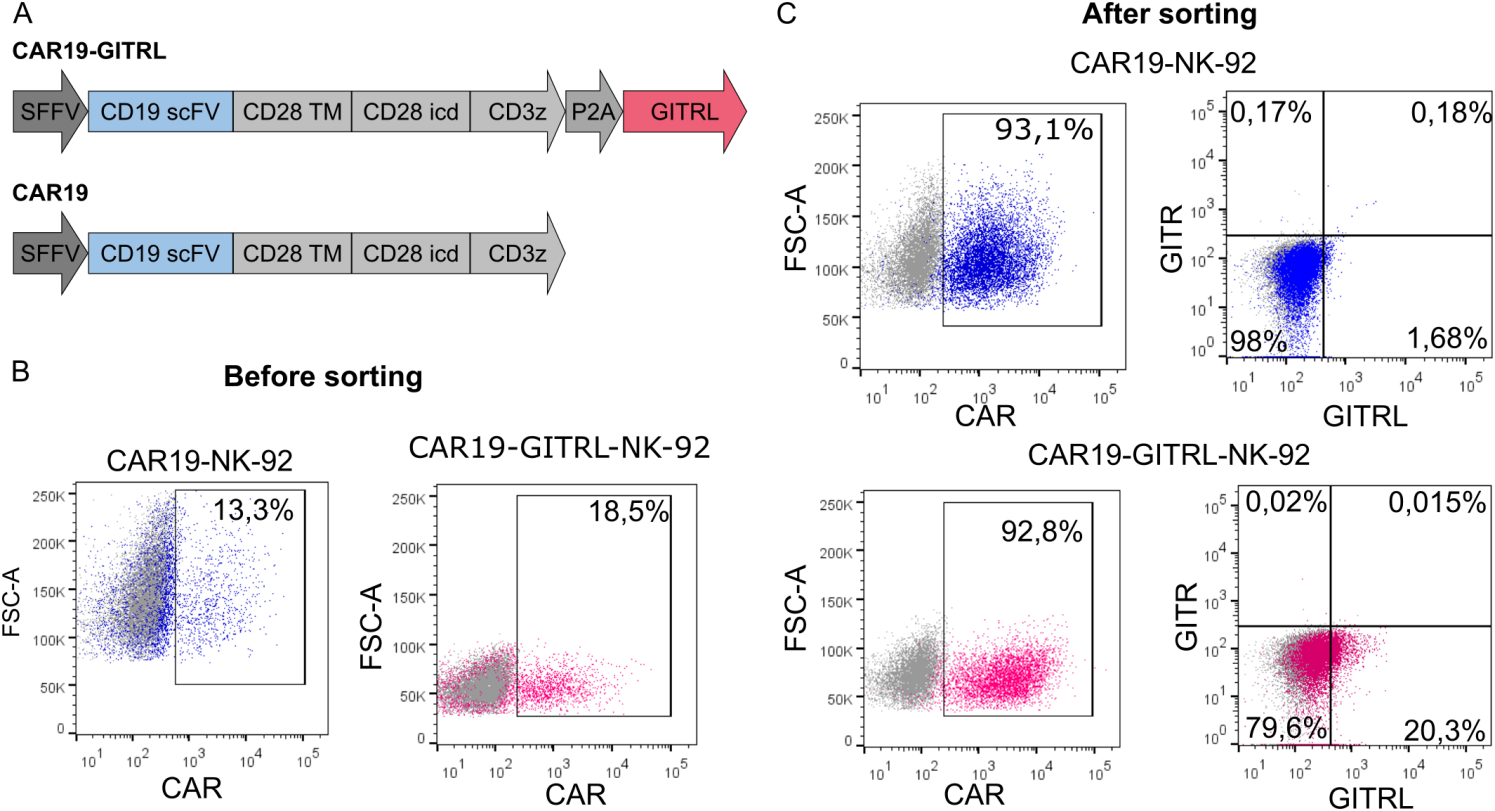
Generation of CAR19-NK-92 and CAR19-GITRL-NK-92 cells. **(A)** SFFV-driven CAR19 and CAR19-GITRL constructs: FMC63 anti-CD19 scFv linked to CD28 transmembrane and CD28/CD3ζ signaling domains; CAR19-GITRL includes a P2A sequence followed by GITRL, whereas CAR19 contains a stop codon. **(B)** NK-92 cells were transduced with VSV-G–pseudotyped lentiviral particles (MOI 10) in the presence of polybrene (8 μg/mL) and BX795 (16 μM); CAR expression was assessed by flow cytometry. **(C)** CAR-positive cells were enriched by FACS (FACSAria Fusion), and CAR, GITR, and GITRL expression were quantified by flow cytometry.

We engineered a fourth-generation anti-CD19 CAR construct in a lentiviral vector that co-expresses GITRL under the control of the spleen focus-forming virus (SFFV) promoter. The CAR construct comprises the FMC63-scFv for CD19 recognition, an IgG-derived hinge, a CD28 transmembrane domain, and signaling domains for CD28 and CD3ζ. A self-cleaving P2A sequence was inserted after the CAR sequence to enable GITRL co-expression (CAR19-GITRL). A second-generation CAR construct lacking the GITRL sequence was designed as a control (CAR19) (**Figure 1A**). Transduction of the NK-92 cell line in the presence of polybrene and BX795 generated CAR19-NK-92 and CAR19-GITRL-NK-92 cells, with CAR expression levels of 13% and 18%, respectively (**Figure 1B**). To enrich the CAR-positive population, we performed cell sorting, and a CAR expression over 90% was achieved for both populations (**Figure 1C**). Although CAR expression exceeded 90% after sorting, GITRL surface expression was detected in a subset of CAR-positive cells, consistent with post-translational regulation and/or differential P2A cleavage efficiency (24) (**Figure 1C**). In addition, GITR was not detected on NK-92 cells, indicating that GITRL is unlikely to act through an autocrine GITR–GITRL axis in this model.

### 3.2 CAR19-GITRL-NK-92 cells display enhanced proliferation and metabolic activity

**Figure 2:**
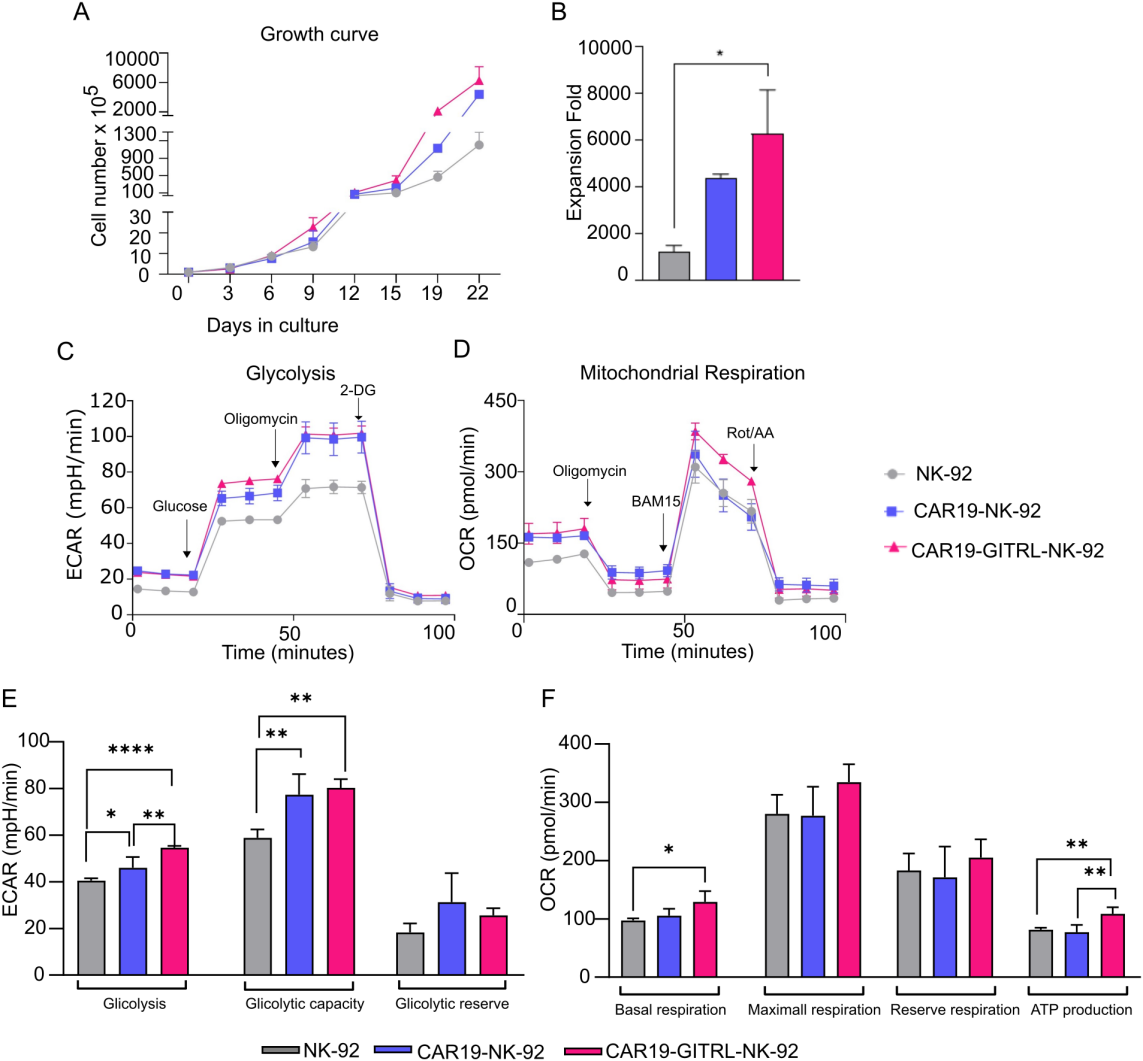
CAR expressing cells demonstrate enhancement in growth and metabolic activity. **(A)** NK-92, CAR19-NK-92 and CAR19-GITRL-NK-92 were plated at 1 x 105 cells/mL and cultivated for 22 days. The cells were counted every 3–4 days using the Neubauer camera. n=2 ± s.d; **(B)** Fold expansion analysis at the end of the 22-day cultivation period. ANOVA one-way with Tukey *post hoc*. *p<0,05. NK-92, CAR19-NK-92 and CAR19-GITRL-NK-92 were plated at 2 x 105 cells/mL and analyzed for **(C)** Glycolysis through Extracellular acidification rate (ECAR) or **(D)** Oxidative Phosphorylation through oxygen consumption rate (OCR) **(E)** Analysis of glycolysis, glycolytic capacity and glycolytic reserve. **(F)** Analysis of basal respiration, maximal respiration, reserve respiration and ATP production. n=4 ± s.d. ANOVA one-way with Tukey *post hoc*. *p<0,05; **p<0,01; ****p<0,0001.

To determine whether CAR or GITRL expression influences NK-cell expansion under standardized culture conditions, cells were maintained at 1 × 10^5^ cells/mL and cultured for 22 days. At the end of a 22-day period, CAR19-GITRL–NK-92 cells expanded to 6.2×10^8^ cells, exceeding both CAR19–NK-92 (4.3×10^8^) and NK-92 cells (1.2×10^8^) (**Figure 2A**). Notably, CAR19-GITRL-NK-92 cells demonstrated a significantly superior expansion fold compared to NK-92 cells (p=0.0376) (**Figure 2B**). Based on the log-phase window (days 3–9), CAR19-GITRL–NK-92 demonstrated the fastest growth kinetics, with a minimal doubling time of 46 hours, in contrast to 62 hours for CAR19–NK-92 and 70 hours for NK-92.

Given that metabolism influences both proliferation and cytotoxic capacity of NK cells (25–27), the bioenergetic profile was evaluated. Glycolytic activity was detected by the extracellular acidification rate (ECAR), and mitochondrial respiration was measured by the oxygen consumption rate (OCR). We observed increased ECAR for both CAR19-NK-92 cells and CAR19-GITRL-NK-92, indicating an enhanced glycolysis metabolism (**Figure 2C**). Further analysis of ECAR data demonstrated that CAR19-GITRL-NK-92 cells exhibited higher glycolytic metabolism compared to NK-92 and CAR19-NK-92 cells. The glycolytic capacity of CAR19-NK-92 and CAR19-GITRL-NK-92 was higher than that of untransduced cells, while for glycolytic reserve, no difference was observed (**Figure 2E**). Mitochondrial respiration analyses revealed increased values for CAR cells, particularly in basal respiration (**Figure 2D**). OCR analysis indicated that only CAR19-GITRL-NK-92 cells exhibited significantly higher basal respiration compared with NK-92 cells. Additionally, CAR19-GITRL-NK-92 cells showed significantly greater ATP production among all the cells. No significant differences were observed in maximal respiration and reserve capacity; however, GITRL co-expressing cells showed a tendency to higher values (**Figure 2F**). Collectively, these findings indicate that CAR19-GITRL–NK-92 cells display an enhanced metabolic profile.

### 3.3 CAR-engineered cells displayed increased cytotoxicity against CD19^+^ targets, accompanied by higher degranulation and cytokine release

**Figure 3:**
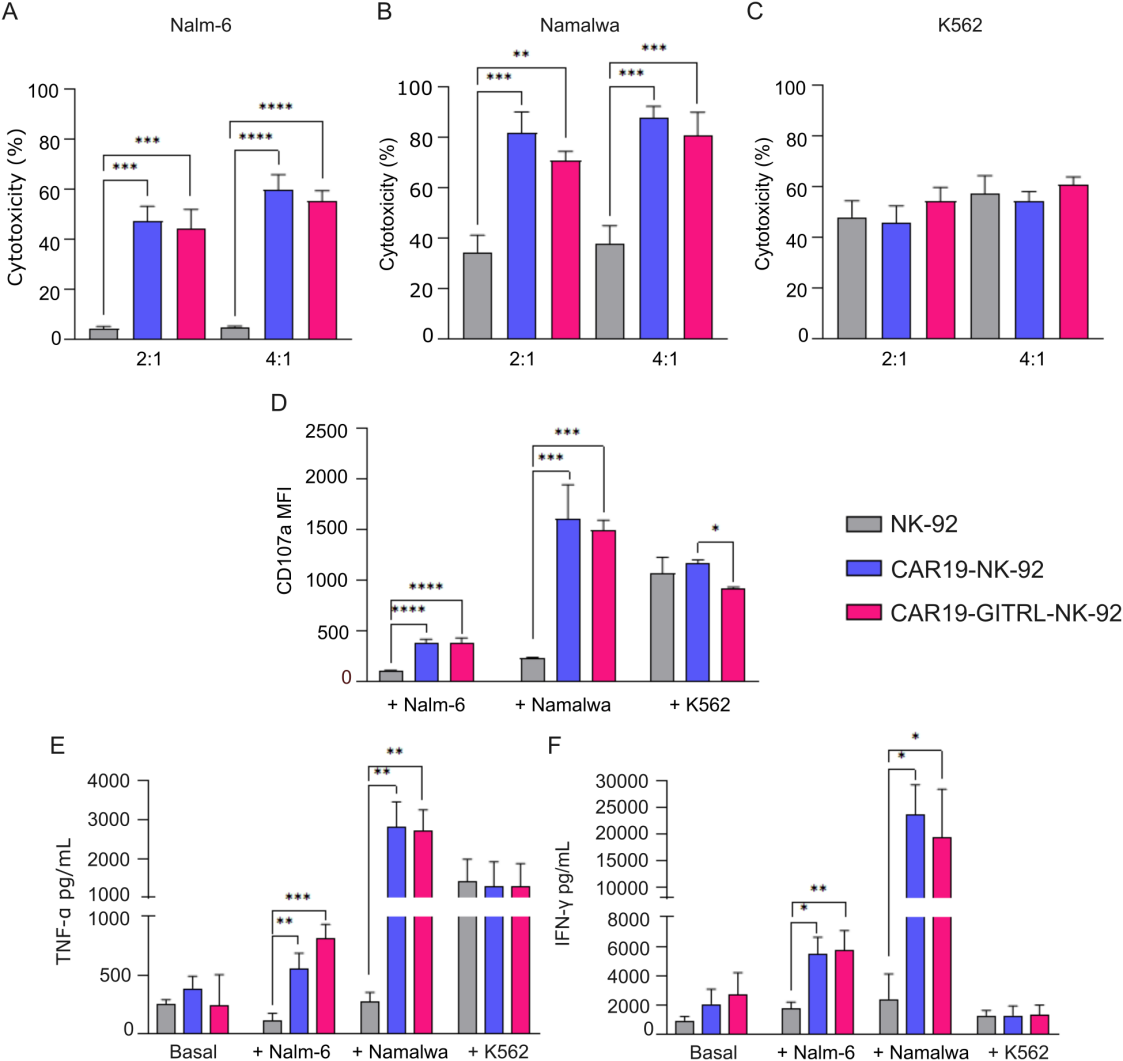
CAR expression increases cytotoxicity, degranulation and cytokine secretion **(A–C)** NK-92, CAR19-NK-92 and CAR19-GITRL-NK-92 were cocultured at 2:1 and 4:1 E:T with the tumor cells Nalm-6, Namalwa, or K562 stained with PKH67. After 5h of coculture, cells were stained with DAPI and analyzed by flow cytometry. **(D)** Degranulation of NK-92, CAR19-NK-92 and CAR19-GITRL-NK-92 after 5h coculture with tumor cells Nalm-6, Namalwa, or K562 at 2:1 E:T. Cells were stained with CD107a and CD56 antibodies, followed by flow cytometry analysis. **(E, F)** TNF-a and IFN-g production by NK-92, CAR19-NK-92, and CAR19-GITRL-NK-92 after co-culture with tumor cells Nalm-6, Namalwa, or K562 for 5h at 2:1 E:T. Cytokine levels in supernatants were analyzed using ELISA. The values in pg/mL were calculated based on standard curves. n=3 ± s.d. ANOVA one-way with Tukey post hoc. *p<0,05; **p<0,01; *** p<0,001; **** p<0,0001.

To assess the cytotoxic potential of CAR-NK-92 cells, we performed co-culture of CAR19-NK-92 and CAR19-GITRL-NK-92 cells with CD19^+^ cell lines Nalm-6 and Namalwa, while the CD19^−^ cell line K562 was included as a negative control (**Figures 3A–C**). After co-culture, both CAR-NK-92 cells exhibited increased cytotoxicity against target cells Nalm-6 and Namalwa than NK-92 cells. The CAR constructs similarly enhanced cytotoxicity under the tested conditions with CD19+ cell lines. No significant variation was observed among the effector cells against the control cell line, supporting the antigen-specific activity of the CAR constructs (**Figure 3C**).

To further characterize the cytotoxic effect, we evaluated the expression of the degranulation marker CD107a. A significant increase in degranulation was observed in CAR19-NK-92 and CAR19-GITRL-NK-92 cells compared to non-transduced NK-92 when cocultured with CD19^+^ target cells (**Figure 3D**). No differences were detected relative to NK-92 cells when co-cultured with the control K562 cell line. Moreover, CAR19-GITRL-NK-92 had lower degranulation against control cells than CAR19-NK-92 cells. The release of cytokines was also measured to investigate the mechanisms involved in the antitumor response (**Figures 3E, F**). CAR-engineered cells showed increased TNF-α and IFN-γ production in response to CD19^+^ targets, while no difference was observed toward K562 or under basal conditions.

### 3.4 CAR19-GITRL-NK-92 cells show upregulated immune pathways compared to CAR19-NK-92

**Figure 4:**
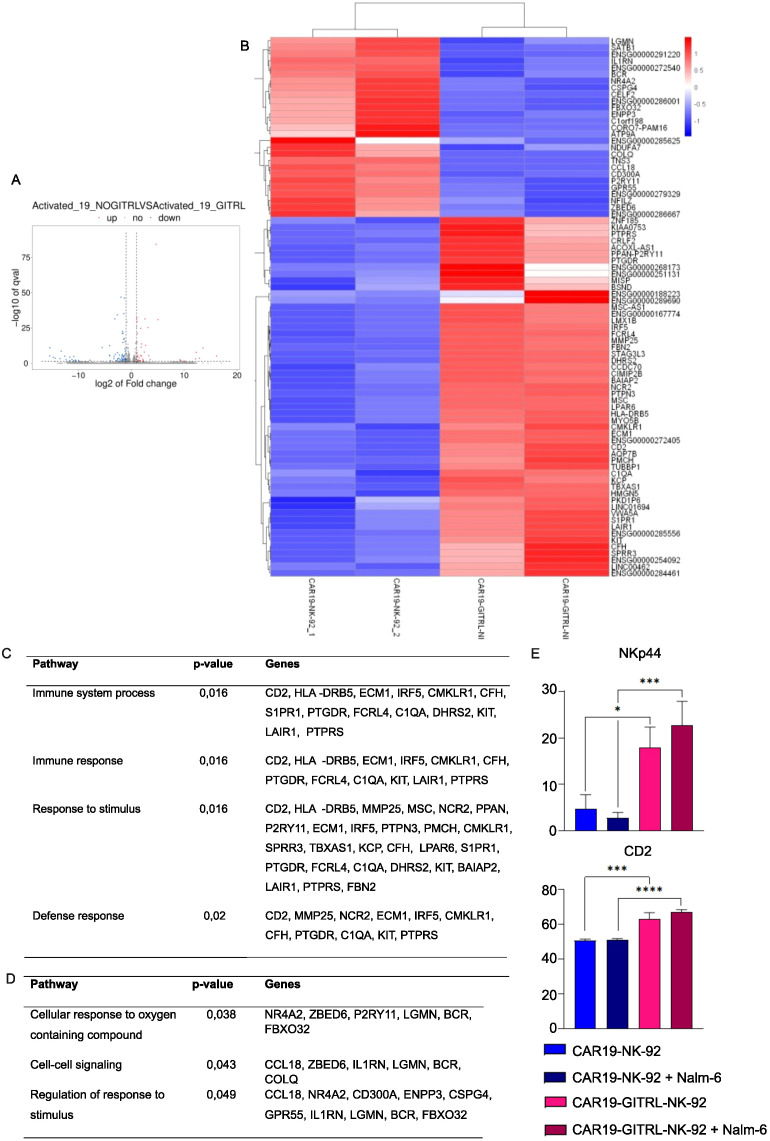
RNA-seq analysis identified differentially expressed genes between CAR cells, revealing enrichment proliferative and effector pathways in CAR19-GITRL-NK-92 cells. **(A)** Volcano plot showing the differential gene expression between CAR19-NK-92 and CAR19-GITRL-NK-92 cells, with the log_2_ fold change (FC) plotted against the significance value (-log_10_ p-value). **(B)** Heatmap of differentially expressed genes, clustered according to expression levels. Distinct clusters are formed among the CAR19-NK-92 and CAR19-GITRL-NK-92 replicates, indicating differences in transcriptional profiles. The color intensity reflects the relative levels of gene expression. (n = 2 biological replicates per group). **(C)** Upregulated pathways in CAR19-GITRL-NK-92 cells after co-culture with Nalm-6 cells. **(D)** Downregulated pathways in CAR19-GITRL-NK-92 cells after co-culture with Nalm-6 cells **(E)** Expression of (i) NKp44 and (ii) CD2 assessed by flow cytometry in CAR19-NK-92 and CAR19-GITRL-NK-92 cells before and after co-culture with Nalm-6 cells during 24h at 4:1 ratio. n=3 ± s.d. ANOVA one-way with Tukey *post hoc*. *p<0,05; *** p<0,001; **** p<0,0001.

To deepen the understanding of the molecular mechanisms stimulated by CAR activation, RNA sequencing (RNA-Seq) analysis was performed on CAR19-NK-92 and CAR19-GITRL-NK-92 cells after co-culture with Nalm-6 cells. This analysis allowed the comparison of gene expression profiles between the two CAR constructs, facilitating the exploration of possible different mechanisms of action. The volcano plot depicts the differential gene expression between CAR19-NK-92 and CAR19-GITRL-NK-92 (**Figure 4A**). For CAR19-GITRL-NK-92, we identified 54 upregulated genes and 34 downregulated genes compared to CAR19-NK-92 cells, showing contrasting signatures (**Figure 4B**).

These gene sets were subjected to enrichment analysis using g:Profiler to identify significantly enriched pathways (**Figure 4C**). Among the upregulated genes in CAR19-GITRL-NK-92 cells, we identified genes related to immune system processes, immune response, and response to stimuli. A greater number of genes could be identified as involved in response to stimuli. Among them, we highlight the *NCR2* gene that codes for the NK activating receptor NKp44. We also identified CMKLR1, associated with NK cell chemotaxis, IRF5, which is involved in response to IFN and the tyrosine-kinase receptor KIT, which supports NK proliferation and survival. PTPN3, a tyrosine kinase associated with proliferation and cytotoxicity, which is under the control of the NF-kB pathway, is also upregulated. For the list of downregulated genes, those associated with the regulation of response to stimuli stand out. Some of the genes with decreased expression include CD300A, an inhibitory receptor; FBXO32, an E3 ubiquitin ligase involved in protein turnover regulation; and IL1RN, the interleukin-1 receptor antagonist that inhibits IL-1 signaling. To validate the RNA-seq findings, we assessed the surface expression of NKp44, the receptor encoded by *NCR2*, and CD2 by flow cytometry. Consistent with the transcriptional profile, CAR19-GITRL-NK-92 cells showed higher expression of both NKp44 and CD2 than CAR19-NK-92 cells, both at baseline and after coculture with Nalm-6 target cells. These data support the RNA-seq results and indicate that GITRL expression is associated with increased expression of NK activation and costimulatory markers (**Figure 4E**).

### 3.5 Antileukemic activity of CAR-GITRL-NK-92 cells in a B-ALL *in vivo* model

**Figure 5:**
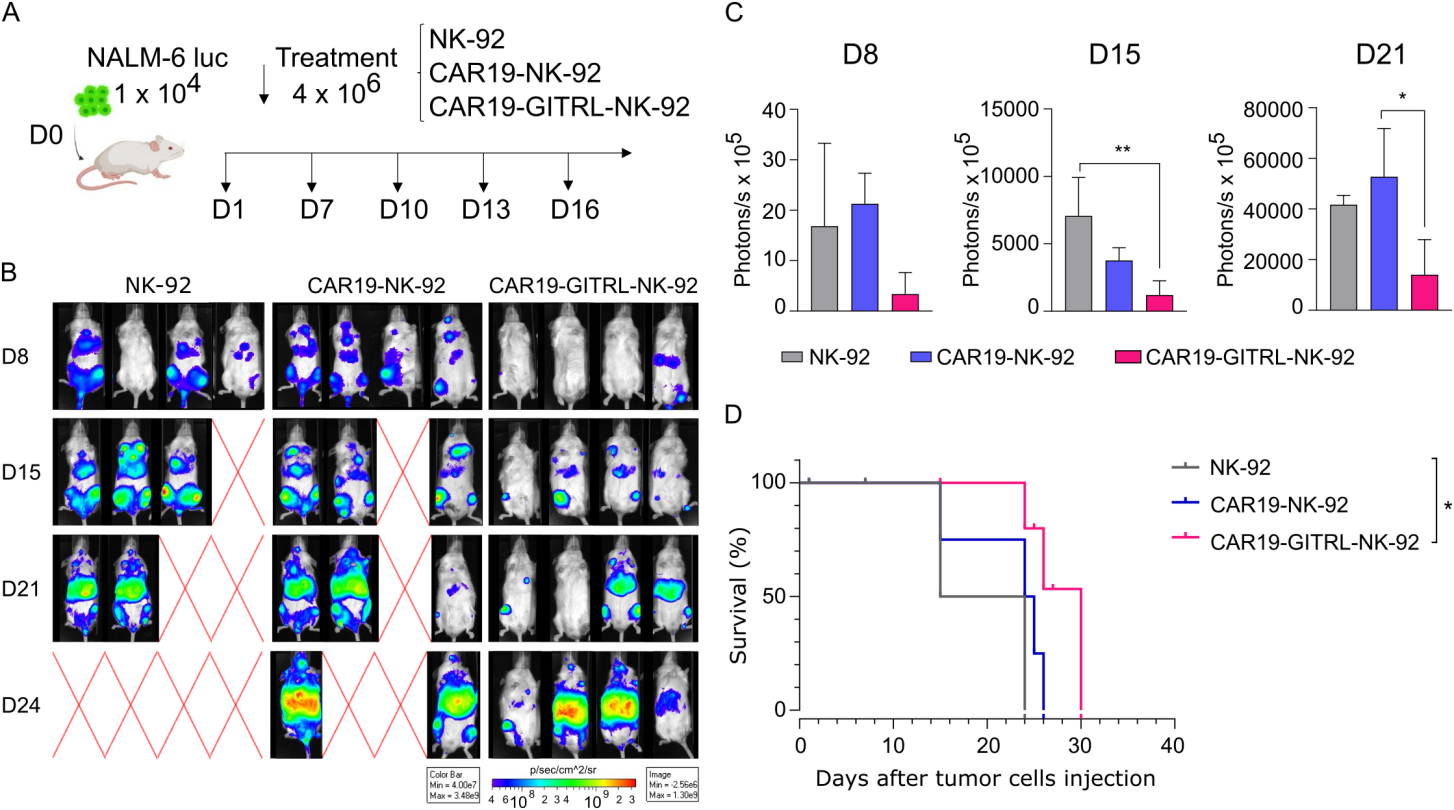
*In vivo* design and improved outcomes with CAR19-GITRL-NK-92 treatment. **(A)** 1×10^4^ Nalm-6-luc cells were infused intravenously on day 0, followed by 5 doses of 4×10^6^ NK-92, CAR19-NK-92, or CAR19-GITRL-NK-92 cells. **(B)** Bioluminescence images were obtained after intraperitoneal injection of luciferin on days 8, 15, 21, and 24. **(C)** Graph showing bioluminescence intensity (photons/s) on days 8, 15, and 21. n=4 ± s.d. One-way ANOVA with Tukey *post hoc*. *p<0.05; **p<0.01. **(D)** Survival curve of animals treated with genetically modified and unmodified NK-92 cells. Log-rank test.

Next, we evaluated the efficacy of CAR cells *in vivo* using a B-ALL xenograft model in NSG mice with Nalm-6 luciferase-expressing cells (Nalm-6-luc). Nalm-6 was chosen due to its resistance to wild-type NK-92 cells. Animals were intravenously injected with 1 × 10^4^ Nalm-6 luc cells (Day 0). Then, mice were randomly assigned to treatment groups and received 5 doses of 4 x 10^6^ NK-92, CAR19-NK-92, or CAR19-GITRL-NK-92 cells (**Figure 5A**). Throughout the experiment, bioluminescence was quantified to evaluate tumor growth. On day 8, no significant difference was observed among the groups, however, treatment with CAR19-GITRL-NK-92 cells showed a trend for decreased tumor development. As the follow-up continued, on days 15 and 21, we observed significantly higher control of tumor growth with CAR19-GITRL-NK-92 treatment (**Figures 5B, C**). By day 24, all the animals from the NK-92 cells treatment were deceased. As shown in **Figure 5C**, animals treated with CAR19-GITRL-NK-92 exhibited a delay in disease progression. However, once the treatment was interrupted, tumor growth resumed. The survival curve revealed a significant difference among the groups, with CAR19-GITRL-NK-92 treatment achieving the longest survival (**Figure 5D**). Collectively, these findings suggest that CAR19-GITRL-NK-92 treatment can modulate tumor progression.

### 3.6 CAR19-GITRL enhances cytotoxic function of primary NK cells against CD19^+^ targets

We next evaluated the CAR19-GITRL vector in primary NK cells isolated from peripheral blood (PB-NK) to confirm its translational potential. PB-NK cells present distinct biological characteristics compared to NK-92 cells, including preservation of CD16 expression, a non-transformed primary-cell phenotype, and greater physiological relevance for evaluating CAR-NK function and interactions with immune regulatory cells. In this context, the use of PB-NK cells allowed us to assess CAR19-GITRL activity in a model that more closely reflects clinically relevant NK-cell biology, including potential interactions between GITRL-expressing CAR-NK cells and GITR-expressing immune cells. In this context, we first investigated whether CAR19-GITRL could enhance the activity of primary NK cells against CD19^+^ target cells.

**Figure 6:**
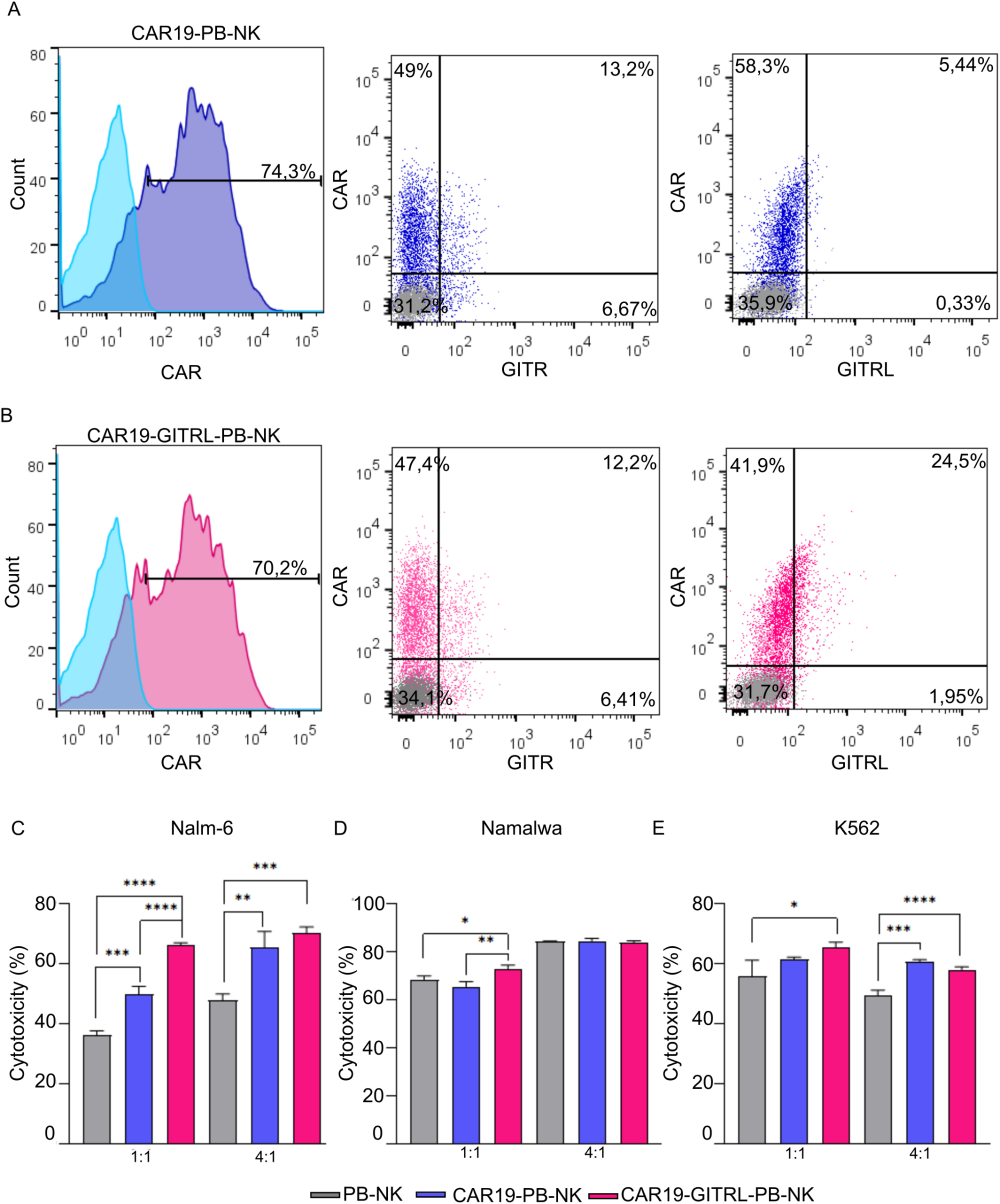
CAR-GITRL-PB-NK cells display increased cytotoxicity. **(A)** flow cytometry analysis of CAR19-PB-NK cells transduced with BaEVRless-enveloped lentivirus, showing: (i) histogram of CAR expression, (ii) CAR x GITR dot plot, and (iii) CAR x GITRL dot plot. **(B)** flow cytometry analysis of CAR19-GITRL-PB-NK cells transduced with BaEVRless-enveloped lentivirus, showing: (i) histogram of CAR expression, (ii) CAR x GITR dot plot, and (iii) CAR x GITRL dot plot. **(C–E)** PB-NK, CAR19-PB-NK and CAR19-GITRL-PB-NK were cocultured at 1:1 and 4:1 E:T with the tumor cells Nalm-6, Namalwa or K562 stained with PKH67. After 5h of coculture, cells were stained with DAPI and analyzed by flow cytometry. Bars represent mean ± s.d. of 3 technical replicates from one donor. ANOVA one-way with Tukey. *p<0.05; **p<0.01; *** p<0,001; **** p<0,0001.

BaEVRless-pseudotyped lentiviruses were used for CAR expression in PB-NK cells. CAR expression levels of 76% and 70% were obtained for CAR19-PB-NK and CAR19-GITRL-PB-NK cells, respectively, with GITRL expression reaching 24.5%. For both cell populations, expression of the receptor GITR was detected in 20% of the cells (**Figures 6A, B**). CAR19-GITRL-PB-NK demonstrated increased cytotoxic activity compared to both CAR-19-PB-NK and PB-NK at a 1:1 ratio against CD19 positive cells Nalm-6 and Namalwa. PB-NK cells demonstrated an intrinsic cytotoxicity against tumor cells, which concealed differences between CAR cells at 4:1 ratio. For Nalm-6 cells, CAR19-PB-NK (p<0,001) and CAR19-GITRL-PB-NK (p<0,0001) had significantly higher activity than PB-NK cells. On the other hand, against Namalwa cells, only CAR19-GITRL-PB-NK cells were more cytotoxic than PB-NK at 4:1 ratio, however these cells showed to be highly responsive to PB-NK cells, comparable to what is observed with NK-92 cells. In coculture with control tumor cells, K562, CAR cells also exhibited higher cytotoxicity than PB-NK cells, indicating a more activated state (p<0,001; p<0,0001) (**Figure 6C**).

### 3.7 GITRL co-expression shows immunomodulatory potential in CAR cells

**Figure 7:**
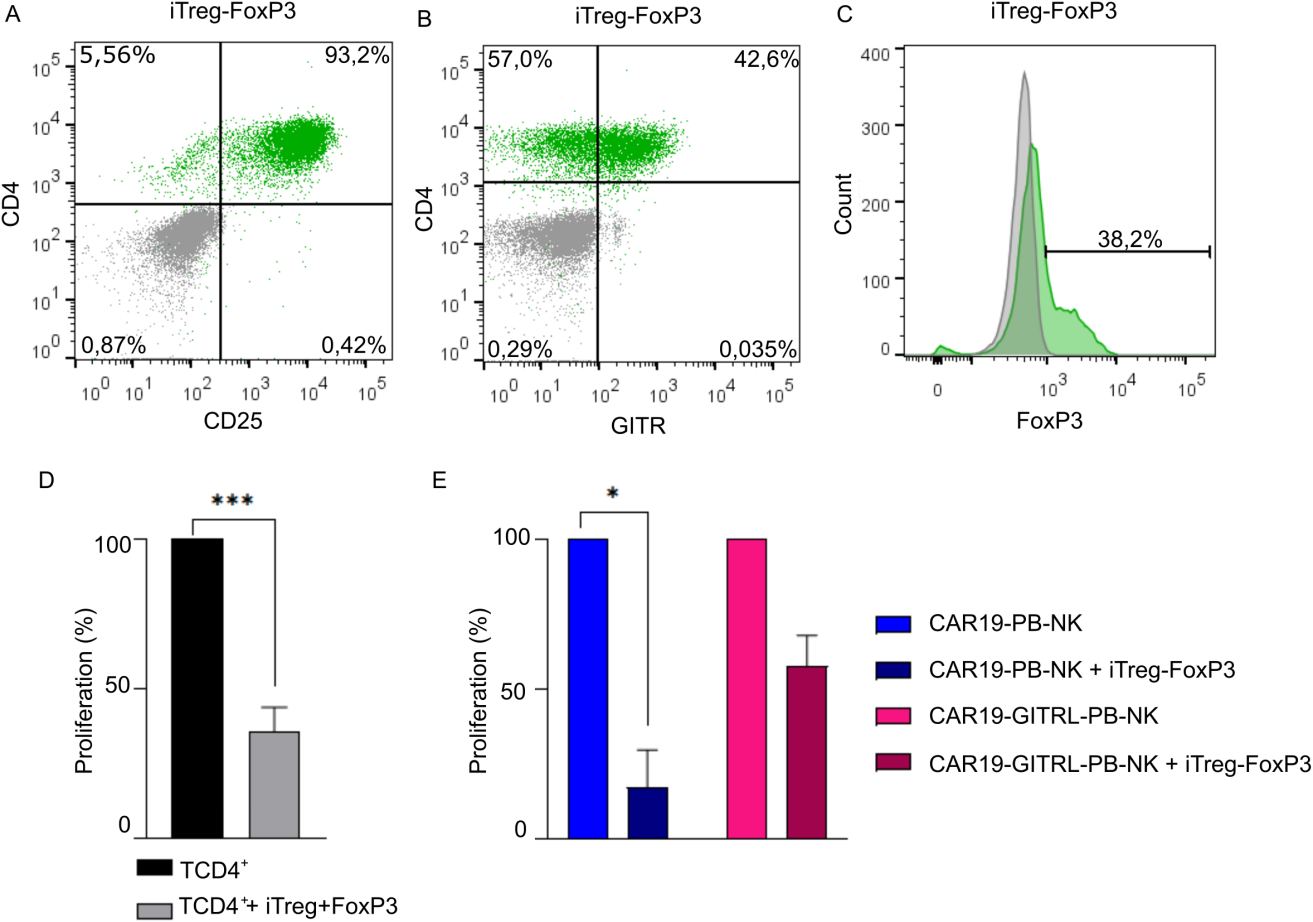
Production of iTreg-FoxP3 cells and their modulation by GITRL co-expression in CAR cells. **(A)** Expression of CD4 and CD25 in T CD4+ cells transduced with the FoxP3 expression vector, the iTreg-FoxP3 cells. **(B)** Expression of CD4 and GITR in iTreg-FoxP3 cells. **(C)** Nuclear Expression of FoxP3 in iTreg-FoxP3 cells. **(D)** Proliferation of TCD4+ cells in co-culture with iTreg-FoxP3 at 1:1 ratio for 72h. The proliferation of control TCD4+ cells was set as 100%. Data are presented as mean ± s.d. (n = 3). Unpaired two-tail Student’s t test; ***p<0.001. **(E)** CAR19-PB-NK and CAR19-GITRL-PB-NK cells were plated at 1:1 ratio with iTreg-FoxP3 for 48h. The iTreg-FoxP3 cells cultured as control were considered to obtain CAR cells growth in coculture. The proliferation of control CAR cells was set as 100%. Bars represent mean ± s.d. of 3 technical replicates from one donor. Unpaired two-tail Student’s t test. *p<0.05.

The interaction of GITRL with its receptor GITR on Treg cells results in the inhibition of their suppressive function (17–19). We hypothesized that GITRL expressed by NK cells modified with the CAR19-GITRL vector could engage with Tregs, diminishing its suppressive activity. To evaluate this interaction *in vitro*, we induced the expression of the FoxP3 gene on CD4^+^ T cells to generate iTreg-FoxP3 cells resembling natural Tregs. These cells, with a 38% expression of FoxP3, presented GITR expression and the capacity to inhibit T cell proliferation (**Figures 7A–D**). We cocultured the generated iTreg-FoxP4 with CAR19-PB-NK or CAR19-GITRL-PB-NK cells (**Figures 6A, B**). As shown in **Figure 7E**, CAR19-GITRL-PB-NK cells was less affected by the presence of iTreg-FoxP3 cells compared to CAR19-PB-NK cells. The data suggest that GITRL co-expression can provide a functional protection against Treg inhibition.

## 4 Discussion

Our findings indicate that GITRL co-expression reshapes the metabolic and functional profile of NK cells. CAR19-GITRL-NK-92 cells demonstrated superior expansion capabilities, reaching higher cell numbers with a reduced doubling time compared to CAR19-NK-92 and NK-92 cells. Improved cell expansion is a critical factor in achieving efficient cell product manufacturing within a short production timeframe. Metabolic analyses revealed that CAR-expressing cells exhibited enhanced glycolysis, with CAR19-GITRL-NK-92 displaying the highest activity. Furthermore, these cells showed significantly increased basal respiration and ATP production, indicating enhanced metabolic fitness. The higher metabolic activity may contribute to their increased expansion (25, 28). More importantly, glycolysis and oxidative phosphorylation are directly involved in NK cell production of IFN-γ and granzyme B, key factors in their cytotoxic activity (26, 27).

Both CAR19-NK-92 and CAR19-GITRL-NK-92 cells exhibited enhanced cytotoxicity against CD19^+^ target cells, Nalm-6 and Namalwa, whereas no significant differences were observed against CD19^-^ K562 cells. Degranulation assays revealed elevated CD107a expression in CAR^+^ cells, highlighting the role of cytotoxic granule release in their killing activity. Cytokine release analysis demonstrated increased TNF-α and IFN-γ production in response to CD19^+^ targets (in both CAR19 and CAR19-GITRL), reinforcing the functional advantages conferred by CAR expression. Moreover, TNF-α and IFN-γ release was directly associated with CAR activation, as NK-92 cells maintained baseline cytokine levels in response to Namalwa cells, despite exhibiting high cytotoxic activity.

RNA sequencing revealed distinct gene expression profiles between CAR19-NK-92 and CAR19-GITRL-NK-92 cells post-activation. Upregulated genes in CAR19-GITRL-NK-92 cells were associated with immune responses and NK cell function, including NCR2 (NKp44), CMKLR1 (chemotaxis), IRF5 (IFN response), KIT (proliferation/survival), and PTPN3 (NF-kB-associated cytotoxicity). As NK cells are activated through the combination of activating and inhibitory signals, a higher expression of activating receptors, such as NCR2, can support the natural activity of NK cells, independently of CAR. CMKLR1 is a receptor expressed in NK cells that can mediate NK cell migration via its ligand chemerin. In a murine model, it was observed that NK cells could inhibit melanoma growth once recruited via CMKLR1 by chemerin overexpression (29). The tyrosine kinase c-kit, encoded by the *KIT* gene, promotes NK cell proliferation when combined with IL-2 or IL-15 signaling (30). Moreover, c-kit has been shown to activate ERK and AKT, which are traditionally linked to NK cell proliferation (31). This result corroborates the shorter doubling time observed in CAR19-GITRL-NK-92 cells. The increased expression of IRF5 and PTPN3 is particularly relevant due to their interactions with the NF-κB signaling pathway, which can be secondarily activated following GITR stimulation. IRF5 interacts with RelA, a subunit of NF-κB, while the tyrosine phosphatase PTPN3 is regulated by NF-κB (32, 33). Downregulated genes included CD300A (inhibitory receptor), FBXO32 (protein turnover regulation), and IL1RN (IL-1 signaling inhibition) (34, 35), suggesting a shift towards an enhanced activation state. Validation of selected RNA-Seq data demonstrated that CAR19-GITRL-NK-92 cells exhibit intrinsically higher expression of CD2 and NKp44 even prior activation. NK cells are activated through the combination of activating and inhibitory signals. A higher expression of activating receptors, such as NKp44, can support the natural activity of NK cells against tumors, independently of CAR (36, 37). As for CD2, in addition to its role in NK cell adhesion, it acts as a co-stimulatory molecule potentiating the signaling of receptors such as NKp46 and NKG2C, further supporting NK cell activation (38–40). Notably, the observed expression levels for these receptors remained unchanged after CAR activation. Additional investigation is required to elucidate the mechanisms underlying these differences, particularly given the lack of GITR expression in NK-92 cells, and to explore whether other RNA-seq findings follow a similar pattern.

*In vivo* model with Nalm-6 cells showed that treatment with CAR19-GITRL-NK-92 cells resulted in superior tumor control and increased survival rate compared to CAR19-NK-92 and NK-92 cells. NALM-6 cells display resistance to NK-92-mediated cytotoxicity *in vitro*, which correlates with the reduced survival observed in the NK-92-treated group *in vivo*. In animals treated with CAR19-GITRL-NK-92 cells, on days 15 and 21, a significantly lower tumor burden was observed, however, tumor progression resumed after treatment discontinuation. NK-92 cells are known to have limited *in vivo* persistence (11), which explains this observation and highlights the need for additional strategies to sustain long-term efficacy. Importantly, during *in vitro* cytotoxicity assays, CAR19-NK-92 and CAR19-GITRL-NK-92 cells did not show significant differences. However, the metabolic alterations observed in CAR19-GITRL-NK-92 cells may contribute to their improved performance *in vivo*. Importantly, GITR expression was not detected in NK-92 cells, indicating that the enhanced fitness observed in CAR19-GITRL-NK-92 cells is unlikely to be mediated by a conventional autocrine GITR–GITRL signaling. It is relevant to consider that members of the TNF superfamily can interact with multiple receptors within the TNFR family, rather than functioning exclusively through a single ligand–receptor pair (41). In addition, TNF superfamily ligands may also participate in reverse signaling or establish non-canonical interactions with receptors other than their classical partners (42). Therefore, although the precise mechanism remains to be elucidated, our functional and RNA-seq data support the possibility that GITRL co-expression triggers non-classical signaling in NK-92 cells, which may contribute to their enhanced metabolic and proliferative phenotype. Further mechanistic studies will be required to define the molecular pathways involved.

We next evaluated the CAR constructs in primary PB-NK cells. GITRL co-expression significantly increased cytotoxicity, with the most pronounced effect observed against Nalm-6 targets. Under the more challenging 1:1 effector-to-target (E:T) condition, CAR19-GITRL–PB-NK cells outperformed untransduced PB-NK and CAR19–PB-NK cells. Namalwa cells were readily killed by PB-NK cells, thereby reducing the dynamic range between groups. Nevertheless, at 1:1 E:T, CAR19-GITRL–PB-NK still exhibited the highest activity. Notably, CAR19-GITRL–PB-NK cells also showed increased killing of K562 control cells, suggesting a more activated functional state. In contrast, the lack of increased K562 killing by CAR-engineered NK-92 cells supports the specificity of CAR-mediated targeting and argues against an intrinsic, nonspecific cytotoxic effect of the CAR itself. To assess whether the GITRL expressed by our vector could counteract Treg activity, we implemented a model of inducing Treg phenotype in TCD4+ cells with a FoxP3 expression vector. Previous studies have shown that this method offers a stable FoxP3 expression and supports a durable phenotype (43, 44). CAR19-GITRL vector attenuated iTreg-FoxP3 mediated inhibition of CAR19-GITRL-PB-NK cells, supporting its potential to function in TME (45).

In conclusion, this study demonstrates that GITRL armoring of anti-CD19 CAR-NK cells confers a broad functional enhancement that extends beyond antigen-specific targeting. Transcriptomic profiling revealed that GITRL coexpression further reshaped the gene expression profile toward immune signaling, proliferation, chemotaxis, and reduced inhibitory signaling. These intrinsic improvements translated into superior tumor control and prolonged survival *in vivo*, despite the known limitation of NK-92 persistence. Importantly, the functional advantage of GITRL armoring was particularly pronounced in primary PB-NK cells under stringent effector-to-target conditions and in the context of Treg-mediated suppression, supporting its relevance in immunosuppressive environments. In summary, these findings position CAR19-GITRL-NK cells as a next-generation armored CAR-NK strategy that integrates enhanced metabolic fitness, robust antitumor activity, and partial resistance to regulatory immune suppression, supporting further development of NK-based immunotherapies for B-cell malignancies.

## Data Availability

The datasets presented in this study can be found in online repositories. The names of the repository/repositories and accession number(s) can be found in the article/supplementary material.
